# Climate vulnerability factors for temperature-related respiratory mortality: a nationwide two-stage time-series study from 2008 to 2021

**DOI:** 10.1186/s12940-026-01309-4

**Published:** 2026-05-23

**Authors:** Hsiao-Yu Yang, Shin-Chieh Chen, Hsi-Yun Chang, Yao-Yuan Wang

**Affiliations:** 1 Institute of Environmental and Occupational Health Sciences, National Taiwan University College of Public Health, No. 17 Xuzhou Road, Taipei, 10055 Taiwan; 2 Department of Public Health, National Taiwan University College of Public Health, Taipei, Taiwan; 3 Population Health Research Center, National Taiwan University, Taipei, 100 Taiwan; 4 Department of Environmental and Occupational Medicine, National Taiwan University Hospital, Taipei, 100 Taiwan; 5 Department of Family Medicine, National Taiwan University Hospital Yunlin Branch, Yunlin, 640 Taiwan

**Keywords:** Climate change, Temperature, Vulnerability, Distributed lag non-linear model, Meta-regression, Geographically weighted regression

## Abstract

**Background:**

Taiwan is one of the fastest-warming regions globally. As climate change intensifies, understanding how vulnerability influences health outcomes critical. This study aimed to identify regional vulnerability factors for temperature-related respiratory mortality and effective region-specific adaptation policies.

**Methods:**

A two-stage time-series study was conducted using daily respiratory mortality counts aggregated by county and day. This study employed a distributed lag non-linear model to estimate the temperature-attributable mortality burden from respiratory diseases across all counties and cities in Taiwan. A two-stage meta-analysis was conducted to estimate temperature–mortality associations and quantify cold- and heat-related mortality burdens by county. Meta-regression was used to identify regional vulnerability factors modifying temperature-related mortality risk, and geographically weighted regression (GWR) was applied to characterize the spatial heterogeneity of these effects across counties.

**Results:**

Cold exposure was linked to a higher burden of respiratory disease mortality (attributable fraction [AF]: 2.03%, 95% CI: 1.10–2.95) than heat exposure (AF: 1.02%, 95% CI: 0.65–1.40). For cold-related AFs, higher proportions of Indigenous populations (3.27, 0.79–5.75), low-income populations (2.11, 0.67–3.55), greater population density (2.21, 0.46–3.96), and children (0.98, 0.35–1.61) were significantly associated with increased risk, suggesting vulnerability factors. GWR further showed that hospital bed availability had statistically significant protective effects against cold-related AF in 10 of 19 counties (β = − 5.24 to − 6.78), most pronounced in remote mountainous counties (Hualien, Taitung, Kaohsiung).

**Conclusion:**

Higher proportions of Indigenous populations, low-income population, and children amplify cold-related respiratory mortality. Hospital bed availability confers the strongest protection against cold-related mortality in remote, mountainous counties. Climate adaptation policies for cold-related respiratory health should therefore be tailored to local vulnerability profiles, prioritizing healthcare expansion in geographically remote counties rather than applying uniform investment across all regions.

## Introduction

Chronic respiratory disease was the third leading cause of mortality globally in 2019, and is associated with a substantial burden and cost [1]. Exposure to non-optimal temperatures can increase respiratory health risks. Cold-air inhalation can induce bronchospasm, dehydration of the airway epithelium, and release of inflammatory mediators [2]. In contrast, exposure to high temperatures may induce airway inflammation and elevate pulmonary vascular resistance, potentially resulting in microvascular thrombosis [3]. Identification of vulnerable individuals to these hazardous effects is crucial for the development of effective intervention programs.

Small-area characteristics can influence climate-related health risks. The regional variation in the health impacts of cold and heat is evident; however, the specific regional factors that modify the temperature–respiratory associations remain unclear. A study of 135 U.S. cities found that temperature–mortality effects vary by race, gender, poverty, population density, education, and green or blue spaces [4]. A multi-country analysis also showed that heat-related mortality increases with fine particulate matter (PM_2.5_), population density, GDP, and income inequality, but decreases with more green space [5]. Identifying vulnerability factors is key to guiding climate adaptation policies.

Vulnerability, as defined by the IPCC Sixth Assessment Report, refers to the propensity to be adversely affected and includes both sensitivity to harm and limited capacity to cope and adapt. These factors can moderate the strength of associations between climate exposure and health outcomes [6]. Climate-related vulnerability factors for respiratory diseases remain unclear, limiting the development of regional climate adaptation policies.

This study aims to identify regional vulnerability factors that modify the association between temperature and respiratory mortality, and to provide evidence-based recommendations for regional climate adaptation policies.

## Methods

A two-stage time-series study was conducted utilizing nationwide respiratory mortality records from Taiwan aggregated by county and day to assess the association with temperature exposure. We applied a Distributed Lag Non-Linear Model (DLNM) to estimate the temperature-attributable mortality burden from respiratory diseases across all counties and cities in Taiwan [7]. A two-stage meta-analysis was subsequently conducted to obtain robust estimates of the temperature–mortality association and to quantify cold- and heat-related mortality burdens in each county. To explore the modifying effects of vulnerability factors on this association, this study applied two complementary analytical approaches: meta-regression was used to identify significant regional vulnerability factors, weighting each county’s attributable fraction (AF) by the inverse of its variance to prevent counties with smaller populations or fewer events from distorting overall inference; geographically weighted regression (GWR) was further applied to characterize the spatial heterogeneity of each factor’s effect, estimating county-specific local coefficients to inform locally targeted adaptation policies [8]. 

### Mortality data

The mortality data used in this study were obtained from the National Health Insurance research database, which is managed by the Health and Welfare Data Science Center, Ministry of Health and Welfare. The Multiple Cause of Death dataset provides comprehensive mortality data, covering all registered deaths in Taiwan since 2008. This study extracted death records from January 1, 2008, to December 31, 2021, and identified deaths from respiratory diseases defined as cases in which any of the first three listed causes of death were coded as J00–J99 under the 10th Revision of the International Classification of Diseases (ICD-10). Based on the date of death, the number of respiratory-related deaths was aggregated by date and administrative region. Throughout this study, “county/city” refers to the top-level administrative division in Taiwan of the 22 such units nationwide, the 19 counties/cities located on the main island of Taiwan were included in the analysis, while the offshore island groups (Penghu, Kinmen, and Lienchiang) were excluded because several vulnerability indicators and kriged air pollution surfaces could not be reliably constructed for their small populations.

### Meteorological data

The meteorological data covered the period from January 1, 2008, to December 31, 2021, and were obtained from the Central Weather Administration’s Climate Data Inquiry Service. For this study, we extracted hourly data on air temperature and relative humidity. To better represent environmental conditions experienced by the general population, only meteorological stations located at elevations below 400 m were included in the analysis to avoid bias from high-altitude stations, which typically record lower temperatures but are in sparsely populated mountainous areas. Metadata for station elevation and location were obtained from the Ministry of Digital Affairs – Government Open Data Platform: Basic Information on Meteorological Stations. To obtain a spatially representative daily value for each county/city, we applied a two-step geospatial procedure. First, hourly observations at each station were averaged to daily station-level values using the *timeAverage* function in the R package openair. Second, daily station values were interpolated to a continuous raster surface using ordinary kriging (ArcGIS Spatial Analyst, Interpolation, Kriging), and the spatial mean of the kriged surface within each county polygon was computed using zonal statistics (ArcGIS Spatial Analyst, Zonal, Zonal Statistics as Table). This kriging–zonal statistics approach has been applied in previous Taiwan-based studies of temperature-related mortality [9].

### Air quality data

Air quality data were obtained from the Environmental Protection Administration’s Air Quality Monitoring Network (Supplementary Fig. 1). We obtained hourly data of carbon monoxide (CO), nitrogen oxides (NOₓ), ozone (O₃), sulfur dioxide (SO₂), and PM₂.₅ from January 1, 2008, to December 31, 2021. To ensure representativeness of air pollution exposure in areas with higher population density, only general (non-industrial) monitoring stations were included. Hourly station observations of CO, NOₓ, O₃, SO₂, and PM₂.₅ were first averaged to daily station-level values, and daily values were then aggregated to the county level using the same ordinary kriging followed by zonal statistics procedure described in Sect.  2.2, yielding one representative daily value per county for each pollutant.

### Vulnerability factors

The vulnerability factors used in this study were obtained from the “Urban, Rural, and Regional Development Statistical Database” published by the National Development Council (Table 1). This study calculated regional averages of vulnerability factors using available data from 2011 to 2020. To ensure comparability across regions and reduce instability caused by sparse data or limited sample sizes in certain counties, we applied a two-stage analytical approach. In the first stage, we used DLNMs to estimate county-specific cumulative exposure–response relationships between daily mean temperature and respiratory disease mortality. The minimum mortality temperature (MMT) was defined as the temperature associated with the lowest point of the cumulative exposure–response curve for each county or city [10]. To prevent the influence of fewer extreme temperature dates and limited mortality data in certain counties, we excluded temperature values beyond the 5th and 95th percentiles when identifying the MMT [11]. In the second-stage analysis [12], a multivariate meta-analysis was conducted to pool region-specific estimates and derive an overall exposure–response function. A unified MMT was identified from the pooled curve and used as the common reference temperature for calculating attributable numbers (ANs) and attributable fractions (AFs) in each county or city [13]. 

**Table 1:** Definitions and formulas for vulnerability factors

Vulnerability Factor	Definition	Formula	Reference
Proportion of children (%)	This indicator represents the percentage of individuals aged 0–14 years within the registered population.	(Number of individuals aged 0–14 ÷ Total registered population) × 100%	[14]
Proportion of elderly population (%)	This indicator reflects individuals aged 65 years and above in the registered population.	(Number of individuals aged ≥ 65 ÷ Total registered population) × 100%	[5, 15]
Proportion of Indigenous population (%)	This indicator reflects the percentage of Indigenous individuals in the registered population.	(Number of Indigenous individuals ÷ Total registered population) × 100%	[16]
Proportion of single-parent households (%)	This indicator reflects the proportion of households headed by a single parent.	(Number of single-parent households ÷ Total registered households) × 100%	
Proportion of low-income population (%)	This indicator reflects the percentage of individuals identified as low-income among the registered population.	(Number of low-income individuals ÷ Total registered population) × 100%	[17]
Population density (persons/km²)	This indicator represents the number of people living per square kilometer of land area.	(Total population ÷ Land area in square kilometers)	[5]
Average green space area (hectares/10,000 persons)	This indicator represents the total area of parks, green spaces, children’s playgrounds, sports facilities, and public plazas per 10,000 registered residents.	(Total area of parks, green spaces, playgrounds, sports venues, and plazas ÷ Total registered population) × 10,000	[5, 15]
Number of hospital beds per 10,000 people	This indicator represents the number of hospital beds available per 10,000 registered residents.	(Number of hospital beds ÷ Total registered population) × 10,000	[5]
Social welfare expenditure ratio (%)	This indicator represents the proportion of total government expenditure allocated to social welfare.	(Social welfare expenditure ÷ Total government expenditure) × 100%	

### Stage 1 county-specific DLNM modeling

We first fitted county-specific quasi-Poisson regression models to estimate the association between daily mean temperature and daily death counts. The exposure–response dimension was modeled using a B-spline with 4 degrees of freedom (df) and internal knots placed at the 10th, 75th, and 90th percentiles of county-specific temperature distributions [5, 18, 19]. The lag–response dimension was modeled using a natural cubic spline with 3 df across a 21-day lag period [19]. The centering value for prediction was initially set as the mean temperature, and later re-centered at the MMT. This study incorporated air pollutants and humidity into the model as confounding factors. Cross-basis functions were specified for daily mean temperature, humidity, and concentrations of PM₂.₅, NOₓ, CO, SO₂, and O₃. A linear function was used to model the concentration–response relationship of air pollutants, while a fourth-degree polynomial function was applied to capture lagged effects, following the approach proposed by Gasparrini et al. [7] Previous studies have shown that air pollution has lagged effects on respiratory mortality, with the greatest impact typically observed between 3 and 7 days [20]. Accordingly, a 7-day lag period was adopted to adequately capture the delayed health effects of air pollutants. To account for seasonal and long-term trends in respiratory mortality, a natural cubic spline function of time was included in the model. For each location 𝑖, the model was specified as:

$$\begin{aligned}\log\lbrack E(Y)\rbrack&\;=\;\alpha\;+\;cb(Temp)\;+\;cb(RH)\;+\;cb(PM_{25})+cb\left(NO_x\right)\;\\&+\;cb(CO)\;+cb\left(SO_2\right)\;+\;cb\left(O_3\right)+ns\left(time_t\right),df=7\\&\times years+\beta_{\mathit1}\cdot holiday\;+\beta_{\mathit2}\cdot dow+\beta_3\cdot doy\end{aligned}$$

Where:

$Y$:daily number of deaths in each location.

$cb(Temp)$: Cross-basis function for daily mean temperature.

$$cb(RH)$$ 

$cb(PM_{2.5})$ , $cb(NOx)$, $cb(CO)$, $cb(SO_2)$, $cb(O_3)$: Cross-basis functions for air pollutants.

*ns*(time, df = 7 × years): Natural cubic spline to adjust for long-term and seasonal trends.

df: degree of freedom.

dow: Day-of-week indicator to control for weekday–weekend effects.

doy: Day-of-year variable to adjust for within-year seasonal patterns (e.g., cold vs. hot seasons).

The study compared DLNM model specifications using different spline functions and degrees of freedom (*df*) for both exposure and lag dimensions. The final model used a B-spline (*df* = 5) for temperature and a natural spline (*df* = 5) for lag. QAIC values for all tested combinations are summarized in Supplementary Table 1.

### Stage 2 multivariate meta-analysis

From each county-specific model, the estimated temperature–mortality association was summarized by a vector of spline coefficients and their corresponding covariance matrix. These were then pooled using a multivariate meta-analysis:

$$y_i\sim N\left(\mu,\;S_i\right),\mu\sim{\mathrm{Fixed}\;\mathrm{Effects}}$$

Where $y_i$ and$S_i$ are the vector of estimated coefficients and their covariance matrix for location $i$, respectively. The pooled estimates $\mu$ were used to reconstruct the national-level cumulative exposure–response curve.

We identified the MMT from the pooled overall curve by selecting the temperature value associated with the lowest relative risk (RR) within the central 5th–95th percentile temperature range. This value was subsequently used as the centering point to improve comparability across regions. The RR were computed with a centering value set to the MMT.

To estimate the ANs and AFs attributable to non-optimal temperatures, we applied the attrdl function developed by Gasparrini, which calculates attributable risk measures based on DLNM [21]. Both ANs and AFs were computed under a backward perspective, using the MMT as the reference. We estimated ANs and AFs for each county based on overall non-optimal temperature exposure, and further separated them into cold (temperatures below the MMT) and heat (temperatures above the MMT). Cold and heat effects were classified into mild cold (5th percentile to MMT), extreme cold (< 5th percentile), mild heat (MMT to 95th percentile), and extreme heat (> 95th percentile) based on county-specific temperature distributions. Random-effects meta-analyses using the restricted maximum-likelihood method were conducted to estimate the pooled effects for each temperature category.

### Meta-regression of vulnerability factors

To assess the impact of regional vulnerability factors, we conducted univariate meta-regression analyses of respiratory mortality AFs under heat and cold exposure. All vulnerability variables were standardized using Z-scores (mean = 0, SD = 1) before analysis to ensure comparability of effect sizes. The resulting regression coefficients were interpreted as the percentage change in AF per one standard deviation increase in the corresponding vulnerability factor [19]. 

### Geographically weighted regression (GWR)

To explore spatial heterogeneity in these associations, we applied geographically weighted regression (GWR), which estimates location-specific coefficients to assess how the strength and direction of associations vary across counties. The resulting GWR coefficients were then mapped to visualize regional variations in effect estimates [22]. A univariate GWR model was fitted for each vulnerability variable using an adaptive bi-square kernel, with bandwidth selection based on AIC optimization. All variables were standardized (z-scores) before analysis to ensure comparability.

### Statistical methods

The analytical procedures included data preprocessing, descriptive statistics, construction of DLNM, estimation of attributable risks, meta-analysis, meta-regression, and GWR. The main R packages used were glm2 for generalized linear modeling, splines for spline construction, dlnm for modeling distributed lag non-linear associations, attrdl for estimating attributable risks, metafor for meta-analysis and meta-regression, and GWmodel for GWR. Two-sided statistical tests were applied throughout, with a p-value < 0.05 considered statistically significant.

### Sensitivity analysis

To identify the optimal lag structure, we tested four temperature–response functions (natural or B-splines with 4 or 5 degrees of freedom) and three lag–response functions (natural splines with 3–5 degrees of freedom). The model with the lowest Quasi-Akaike Information Criterion (QAIC) value was selected.

### Ethics approval

This study was approved by the Research Ethics Committee of National Taiwan University (No. 202305HM147).

## Results

**Fig. 1:**
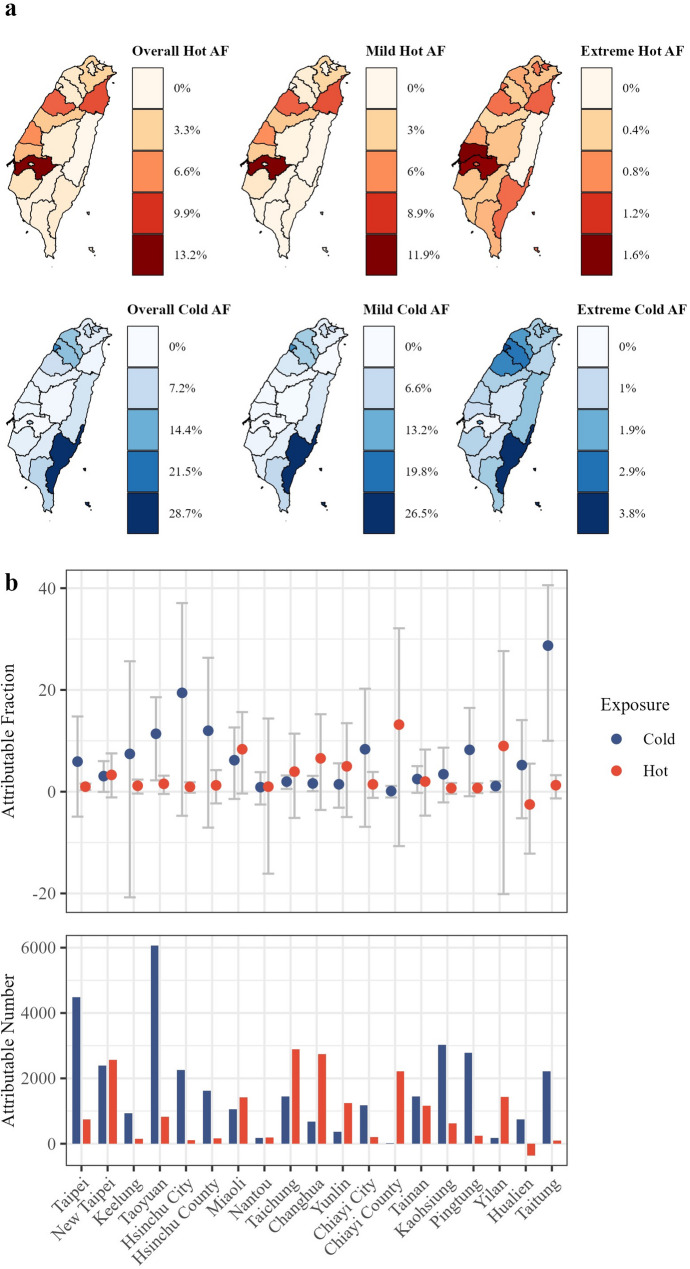
Geographic distribution and quantitative burden of temperature-related respiratory mortality in Taiwan. Legend: (**a**) Maps show the attributable fractions (AFs, %) of respiratory mortality associated with non-optimal temperature exposures in each county, stratified by overall heat and cold, mild heat/cold, and extreme heat/cold. Chiayi County is shown in the darkest brown, indicating the highest hot AF. Taitung County is shown in the darkest blue, indicating the highest cold AF. **b** Bar plots present the AFs and corresponding attributable numbers (ANs) of respiratory deaths for each city/county, grouped by exposure type

This study included 797,898 deaths from respiratory diseases recorded between 2008 and 2021 across 19 counties and cities on the main island of Taiwan. During the study period, average daily temperatures ranged from 18 °C to 24 °C in the cold season (October to March), and from 27 °C to 30 °C in the hot season (from April to September) (Supplementary Table 2). Supplementary Fig. 2 illustrates regional disparities in vulnerability factors across Taiwan. Supplementary Fig. 3 shows the estimated cumulative temperature–mortality associations across all 19 counties and cities. Cold-related risk generally exhibited a gradual increase over longer lags, while heat-related risk rose more steeply over shorter lag periods. Meta-analysis of temperature-related AFs for respiratory mortality showed a reverse J-shaped pattern (Supplementary Fig. 4). Cold exposure below the MMT was associated with a significantly higher burden (AF: 2.03%, 95% CI: 1.10–2.95), primarily driven by extreme cold (AF: 1.27%, 95% CI: 1.02–1.52), while mild cold had no significant effect (AF: 0.00%, 95% CI: −0.00 to 0.01). Heat exposure above the MMT was linked to a smaller but significant burden (AF: 1.02%, 95% CI: 0.65–1.40), with extreme heat contributing more (AF: 0.68%, 95% CI: 0.48–0.88). Mild heat showed no significant association (AF: −0.03%, 95% CI: −0.03 to 0.10). Figure 1 shows the impact of non-optimal temperature on respiratory mortality. Cold temperatures have more influence on respiratory mortality in Taiwan than hot temperatures, with significant extreme-cold AFs observed in 11 of 19 counties. Taitung County exhibited the highest cold-AF at 28.71% (95% CI 12.15–39.78). In contrast, total heat exposure was significant only in Taipei City (0.98% [95% CI 0.38–1.60]) (Supplementary Table 3).

**Fig. 2:**
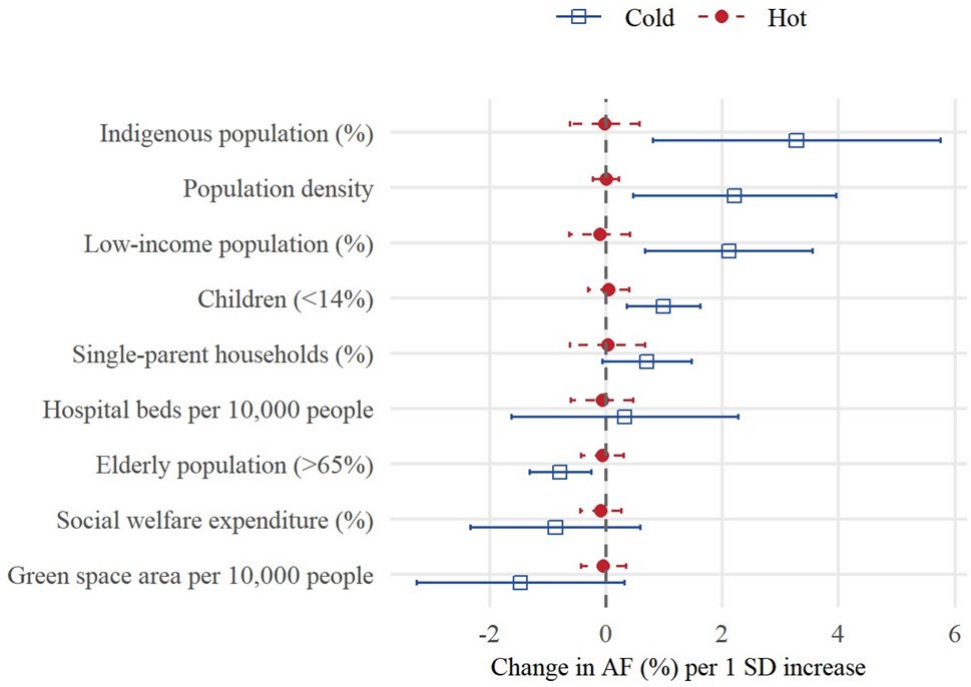
Impact of regional vulnerability factors on respiratory mortality attributable to cold and heat exposure. Legend: The plot shows the change in attributable fraction (AF) of respiratory mortality (%) per 1 standard deviation (SD) increases in each vulnerability factor. Open squares represent the effect estimates for cold-related AF; solid circles represent those for heat-related AF. Error bars indicate 95% confidence intervals. All variableswere standardized before meta-regression analysis

The meta-regression analysis shows that higher proportions of children (estimate: 0.98, 95% CI: 0.35–1.61), Indigenous populations (estimate: 3.27, 95% CI: 0.79–5.75), low-income populations (estimate: 2.11, 95% CI: 0.67–3.55), and greater population density (estimate: 2.21, 95% CI: 0.46–3.96) were significantly associated with increased AFs for cold exposure, whereas a higher proportion of elderly individuals showed a significant inverse association (estimate: − 0.80, 95% CI: − 1.32 to − 0.27). In contrast, higher social welfare expenditure and increased green space were negatively associated with cold-related AFs, suggesting potential protective effects against cold-related respiratory mortality (Fig. 2; Table 2).

**Table 2:** Meta-regression of vulnerability factors and change in AF (%) per 1 SD increase

Vulnerability Factor	Hot Estimate (95% CI)	Cold Estimate (95% CI)
Children (<14 years, %)	0.04 (–0.32, 0.39)	**0.98 (0.35**,** 1.61)**
Elderly population (> 65%)	–0.07 (–0.44, 0.3)	**–0.80 (–1.32**,** –0.27)**
Indigenous population (%)	–0.03 (–0.62, 0.56)	**3.27 (0.79**,** 5.75)**
Single-parent households (%)	0.02 (–0.62, 0.66)	0.70 (–0.07, 1.47)
Low-income population (%)	–0.12 (–0.64, 0.41)	**2.11 (0.67**,** 3.55)**
Green space per 10,000 people	–0.05 (–0.44, 0.34)	–1.48 (–3.26, 0.31)
Social welfare expenditure (%)	–0.10 (–0.45, 0.25)	–0.87 (–2.33, 0.59)
Population density	–0.01 (–0.23, 0.22)	**2.21 (0.46**,** 3.96)**
Hospital beds per 10,000 people	–0.07 (–0.61, 0.46)	0.32 (–1.63, 2.27)

Significant values are highlighted in bold

**Fig. 3:**
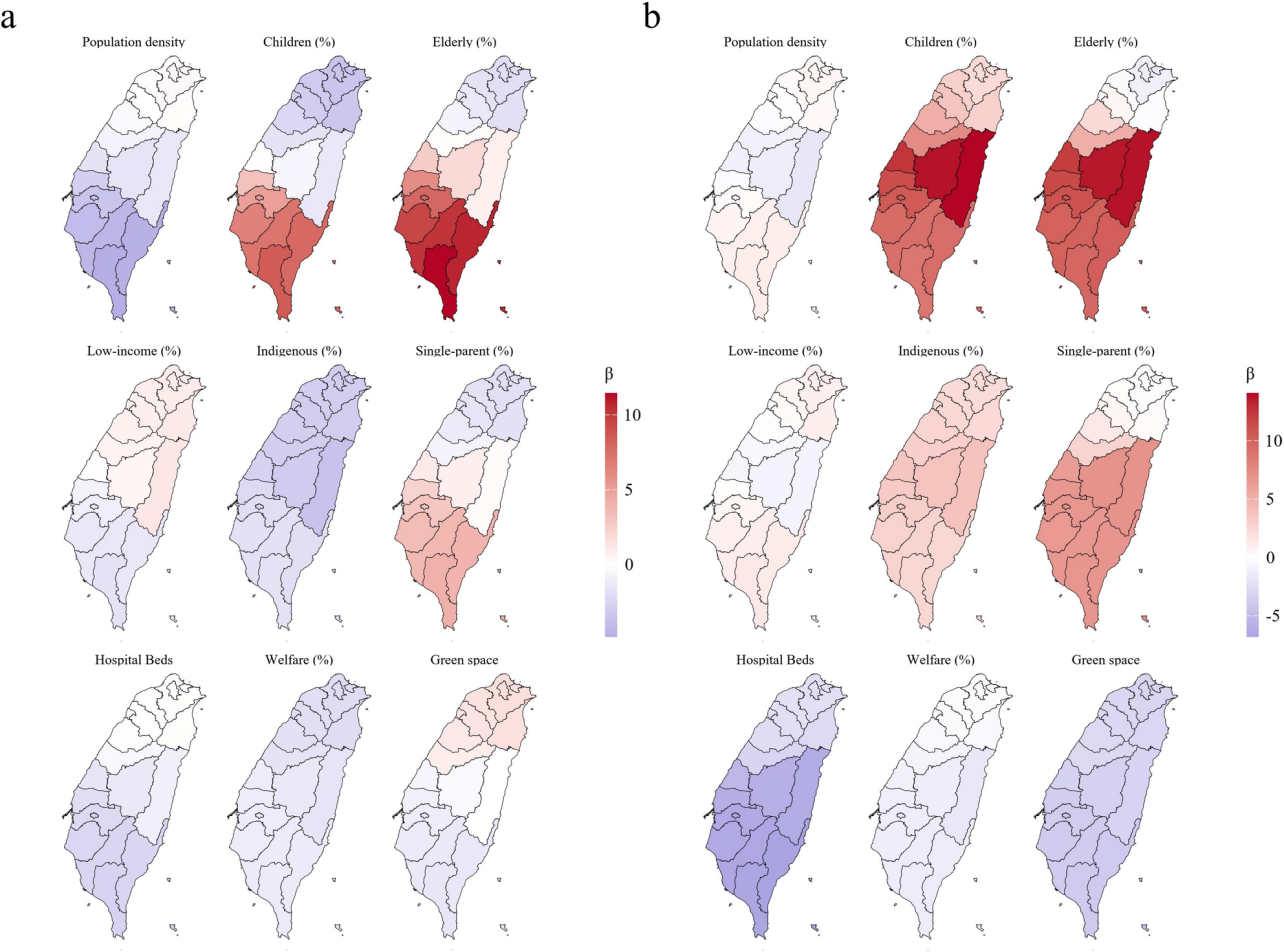
Spatial distributions of local GWR coefficients (β) linking vulnerability factors to heat- and cold-related attributable fractions (AFs) of respiratory mortality in 19 counties of Taiwan. **a** β coefficients for heat-related AFs. **b** β coefficients for cold-related AFs. Colors represent the strength and direction of association. Red = positive association; Blue = negative association

GWR revealed spatial variation in associations between vulnerability factors and temperature-related respiratory mortality (Fig. 3). Most vulnerability-factor associations did not reach local statistical significance, but consistent spatial gradients were observed across regions. For heat-related AF, positive associations with the proportions of children, elderly population, and single-parent households were strongest in southern Taiwan, particularly in Kaohsiung, Pingtung, and Taitung. For example, coefficients for the elderly population ranged from β = +10.20 in Kaohsiung to β = +11.44 in Pingtung, while coefficients for single-parent households ranged from β = +3.70 in Kaohsiung to β = +4.10 in Pingtung (Supplementary Table 4). In contrast, low-income populations showed relatively stronger positive associations in northern and eastern counties, including Keelung (β = +1.00), Yilan (β = +1.08), and Hualien (β = +1.25). For cold-related AF, stronger positive associations with children, elderly populations, and single-parent households were observed in eastern, central, and southern Taiwan. The largest coefficients were identified in Hualien (children: β = +14.07; elderly: β = +13.81), Nantou (children: β = +13.82; elderly: β = +13.67), and Changhua (children: β = +12.51; elderly: β = +12.09) (Supplementary Table 5). Green space and hospital bed availability generally showed protective associations against both heat- and cold-related AFs. Hospital bed availability demonstrated statistically significant protective effects against cold-related AF in 10 of 19 counties, with the strongest effects observed in Taitung (β = − 6.78, *p* = 0.016), Kaohsiung (β = − 6.54, *p* = 0.018), and Hualien (β = − 6.11, *p* = 0.013) (Supplementary Table 5).

## Discussion

To our knowledge, this is the first nationwide study to integrate a two-stage meta-analytic framework with GWR to investigate how regional vulnerability factors modify temperature-related respiratory mortality. The novelty of this approach lies in combining precision-weighted meta-regression—which incorporates the uncertainty of county-level AF estimates from the first-stage DLNM—with GWR, which maps the spatial heterogeneity of each vulnerability factor’s effect across counties. This combined framework simultaneously identifies which sociodemographic and infrastructural factors matter at the national level and whether these effects vary across regions. Notably, GWR revealed that the protective effect of hospital bed availability against cold-related respiratory mortality was concentrated in counties with a large proportion of remote mountainous areas (Hualien, Taitung, and Kaohsiung), where geographic barriers amplify the marginal benefit of additional medical capacity. These findings provide a transferable analytical framework and a scientific basis for designing region-specific climate adaptation policies, prioritizing healthcare resource expansion in geographically constrained regions rather than uniform national investment.

### Cold effect vs. hot effect

This study identified a reverse J-shaped relationship between temperature and respiratory mortality, suggesting that cold temperatures have a greater impact on respiratory mortality than heat. This finding is consistent with a multi-city analysis conducted across 15 European cities, which also reported that the cold effect on mortality was greater in warmer (southern) regions [23]. In respiratory mortality data from the subtropical city of Dongguan, cold exposure contributed to a greater burden than heat exposure [24]. This may suggest that populations living in warmer climates are more acclimated to high temperatures than to cold, making them more vulnerable to cold exposure.

### Indigenous population

This study found that the proportion of the Indigenous population was a significant vulnerability factor for cold-related respiratory mortality, but not for heat. This aligns with findings from Aboriginal populations in the Northern Territory of Australia, where long-term inhabitation in a hot climate without air conditioning has led to physiological acclimation to heat exposure [16]. Cold protection measures—such as improving housing insulation and providing heating support are recommended for regions with high Indigenous populations.

### Low-income population

The low-income population was identified as a significant vulnerability factor for cold-related respiratory mortality. This finding is consistent with a nationwide study in Korea, which reported that heat- and cold-related illnesses were more prevalent among individuals with low income compared to those with higher income [17]. To reduce cold-related health risks in socioeconomically disadvantaged communities, targeted interventions—such as improving housing insulation—are recommended.

### Children (< 14 years)

This study found a positive association between a higher proportion of children and cold-attributable risk. The cold effect was amplified in central and southern Taiwan, suggesting a combined effect with more severe air pollution in these regions. These findings underscore the need for targeted interventions to protect children’s respiratory health during cold spells, particularly in regions with both high child population density and elevated air pollution.

### Single-parent households

This study found that counties with a higher prevalence of single-parent households exhibited increased cold-attributable fractions, particularly in southern Taiwan. The cold-related hazard was further amplified in central and southern Taiwan. While no Taiwan-based study directly examined cold-related AFs by family structure, a spatial analysis identified ‘family fragility’—including single-parent prevalence—as a significant predictor of increased child mortality in southeastern Taiwan [25]. This association could be linked to household-level resource constraints, which may limit the capacity to access health services.

### Elderly population (> 65 years)

Notably, although previous studies have suggested that regions with a higher proportion of elderly individuals may exhibit greater vulnerability during extreme weather events [15], our results showed that the proportion of elderly individuals was not associated with higher AF of respiratory mortality under heat exposure, and was even negatively associated with cold-related AF. The hazardous effect of non-optimal temperature was amplified in central, eastern, and southern Taiwan, but decreased in northern Taiwan, indicating regional heterogeneity.

The finding may reflect regional disparities in healthcare access and socioeconomic status. In northern Taiwan, where healthcare infrastructure is more developed and socioeconomic conditions are more favorable, elderly residents may be better protected against non-optimal temperatures. In contrast, southern Taiwan tends to have a higher proportion of elderly individuals living in areas with less developed healthcare systems and lower socioeconomic status, which may amplify the health impacts of extreme heat. This spatial mismatch between vulnerability and adaptive capacity underscores the need for region-specific interventions that strengthen heat resilience in southern Taiwan.

### Social welfare expenditure

Our study shows that an increase in social welfare expenditure can decrease the burden of respiratory mortality under non-optimal temperatures. Our findings suggest that strengthening local social welfare investment—particularly in vulnerable regions—may serve as an effective public health strategy to mitigate the respiratory health burden under cold exposure.

### Green space area

Different from previous studies showing that higher levels of green space are associated with reduced heat-related mortality [5]. Our study found that a higher proportion of green space could mitigate the impact of cold exposure on respiratory mortality. These findings further support urban greening as an adaptive strategy to reduce the health impacts of extreme temperatures.

### Number of hospital beds per 10,000 people

This study showed that an increase in hospital bed availability can decrease cold-related respiratory mortality. Although greater hospital bed availability is widely believed to improve a population’s resilience to cold-related respiratory deaths, our GWR analysis further showed that hospital bed availability had statistically significant protective effects against cold-related respiratory mortality in 10 of 19 counties, with the strongest effects in Hualien (β = − 6.11, *p* = 0.013), Taitung (β = − 6.78, *p* = 0.016), and Kaohsiung (β = − 6.54, *p* = 0.018). Notably, all three counties contain extensive remote mountainous areas: Hualien and Taitung occupy Taiwan’s eastern coast bounded by the Central Mountain Range and Coastal Range, while Kaohsiung’s eastern districts lie deep in the Central Mountain Range. In these counties, medical facilities are concentrated in coastal or urban townships, and residents of mountainous areas—often Indigenous populations—face long travel distances to access hospital care. Hsieh et al. (2023) documented persistent geographical disparities in healthcare resources across all 368 Taiwanese townships, identifying mountainous and rural townships in eastern and southern Taiwan—including Zhuoxi Township in Hualien and Taoyuan District in Kaohsiung—as areas with significantly higher mortality rates and limited medical infrastructure [26]. Among Indigenous communities in Taiwan’s mountainous regions, qualitative evidence further indicates that long travel distances and difficult terrain substantially constrain access to medical services [27]. The pronounced GWR coefficients therefore suggest that, where healthcare access is inherently constrained by geography, marginal increases in hospital bed availability translate into disproportionately larger reductions in cold-related respiratory mortality. By contrast, hospital beds did not reach statistical significance in northern metropolitan counties (Taipei, New Taipei, Keelung), where healthcare resources are already abundant and additional capacity may yield diminishing returns. These findings underscore that climate adaptation policies for cold-related respiratory health should prioritize healthcare resource expansion in geographically remote and mountainous counties with uneven medical facility distribution, rather than applying uniform investment across all regions.

### Limitation

As an ecological study, this research lacks individual-level exposure data, such as personal activity patterns and microenvironmental exposures, which may limit the precision of the findings. The mortality data were derived from official cause-of-death statistics and aggregated by registered household addresses. However, these addresses may not correspond to actual places of residence—particularly in counties with substantial population out-migration—potentially introducing bias into regional risk estimations. The use of 19 counties as the unit of analysis limited the feasibility of multivariate spatial regression with multiple vulnerability factors. We therefore relied on univariate meta-regression with inverse-variance weighting (which directly incorporates AF precision from the first-stage DLNM) for global vulnerability factor identification, and on GWR for characterizing spatial heterogeneity. This combination provides complementary, methodologically appropriate analyses for our small-area, ecological design.

## Conclusion

This study employed a two-stage DLNM to estimate the burden of respiratory mortality attributable to cold and heat exposure. Meta-regression was conducted to identify vulnerability factors influencing the burden of respiratory diseases. These findings underscore targeted support for households with children, Indigenous and low-income groups. GWR further demonstrated that the protective effect of hospital bed availability against cold-related respiratory mortality was concentrated in geographically remote, mountainous counties. Accordingly, climate adaptation policies for cold-related respiratory health should prioritize healthcare resource expansion in geographically remote and mountainous counties with uneven medical facility distribution, rather than applying uniform investment across all regions. The findings offer a scientific basis for developing region-specific strategies to mitigate the health impacts of extreme temperatures on respiratory health and to enhance climate resilience.

## Data Availability

This research used injury data obtained from the Health and Welfare Data Science Center, Ministry of Health and Welfare. Due to legal restrictions, sharing of the original data is prohibited to protect confidentiality.
